# Comparative study of 11 machine learning algorithms for predicting recurrence risk after atrial fibrillation catheter ablation based on a real-world cohort: a retrospective study

**DOI:** 10.1186/s12872-025-05489-8

**Published:** 2026-01-14

**Authors:** Yicheng Wang, Yi-Ming Peng, Zi-Ao Fan, Xiao-Lin Liao, Hong-Yi Yang, Jian-Quan Chen, Jian-Cheng Zhang

**Affiliations:** 1 Shengli Clinical Medical College of Fujian Medical University, No.134 East Street, Gulou District, Fuzhou, Fujian 350000 PR China; 2 Fuzhou University Affiliated Provincial Hospital, No.134 East Street, Gulou District, Fuzhou, Fujian 350000 PR China; 3 Department of Cardiology, Provincial Clinical Medicine College of Fujian Medical University, Fujian Provincial Hospital, No.134 East Street, Gulou District, Fuzhou, Fujian 350000 PR China

**Keywords:** Atrial fibrillation, Machine learning, Catheter ablation, Prediction model, Recurrence

## Abstract

**Background:**

Atrial fibrillation (AF) is the most common arrhythmia worldwide, with catheter ablation being an effective yet recurrence-prone treatment. Given the limited accuracy of conventional risk scores in identifying patients at high risk of recurrence after catheter ablation, this study sought to develop and validate a machine learning (ML) model for predicting AF recurrence using a wide array of clinical and laboratory variables.

**Methods:**

Of the 438 patients with AF included in this study who underwent catheter ablation between 2016 and 2023. Comprehensive demographic, clinical, echocardiographic, laboratory, medication, and risk score data were collected. The primary endpoint was AF recurrence, defined as documented AF, atrial flutter, or atrial tachycardia ≥ 30 s occurring ≥ 3 months post-procedure. The dataset was randomly divided into training set and validation set in a 6:4 ratio. Univariate and multivariate logistic regression were used to identify independent risk factors for the risk of recurrence after catheter ablation of AF. Eleven ML algorithms were established on the training set—including random forest (RF), gradient boosting machine(GBM), logistic regression (LR), support vector machine(SVM) and XGBoost. Model performance was evaluated using receiver operating characteristic (ROC) curves, precision-recall (PR) curves, and calculating the area under the curve (AUC). A calibration curve assessed the model’s accuracy, while decision curve analysis (DCA) evaluated its clinical applicability. In addition, to avoid overfitting, we conducted an internal validation of best model using Bootstrap. Finally, Shapley additive explanations (SHAP) were employed to interpret the importance of predictor variables.

**Results:**

Of the 438 patients with AF included in this study who underwent catheter ablation, 147 experienced recurrence during follow-up. The median age of the total population was 63 years, with 64 years in the non-recurrence group and 63 years in the recurrence group (*P* = 0.303). The proportion of females was 36.1% in the recurrence group vs. 52.6% in the non-recurrence group (*P* = 0.018). The RF model demonstrated superior performance, achieving an AUC of 0.878 in the training set and 0.925 in the validation set. It also showed excellent calibration (Brier score: 0.186) and clinical utility across a wide risk threshold range. Key predictors included alcohol consumption [OR = 2.12 (1.15–3.91), *P* = 0.017)], fibrin degradation products [FDP, OR = 1.22 (1.02–1.46), *P* = 0.027], and hypertension [OR = 0.47 (0.26–0.85), *P* = 0.012].

**Conclusion:**

An interpretable ML model based on RF accurately predicts AF recurrence post-ablation and outperforms conventional risk scores. This tool may enhance individualized patient counseling, follow-up strategy design, and resource allocation in clinical practice.

## Abstract

Atrial fibrillation is the most common sustained cardiac arrhythmia, affecting an estimated 33 million people worldwide, with a prevalence of approximately 2–4% in the general population and exceeding 10% among those aged over 80 years [1]. The global burden of AF continues to rise, with projections suggesting that the number of affected individuals will more than double by 2050 [2–4]. The pathophysiology of AF is multifactorial and involves a complex interplay of electrical and structural remodeling, atrial fibrosis, inflammation, autonomic nervous system imbalance, and metabolic alterations [5, 6]. These changes not only contribute to AF initiation and maintenance but also increase the risk of thromboembolism, stroke, heart failure, and all-cause mortality, making AF a major public health challenge worldwide [7–10]. Accurate identification of patients at high risk of adverse outcomes, especially recurrence after interventional therapy, is therefore a priority for clinical management.

Catheter ablation has become an established and effective treatment for symptomatic AF, providing superior rhythm control compared with antiarrhythmic drug therapy [11, 12]. Nevertheless, AF recurrence after ablation remains common, with rates ranging from 20% to 50% within the first year depending on patient characteristics and procedural factors [13]. Conventional risk prediction tools—such as clinical risk scores such as CHA₂DS₂-VASc or individual biomarkers—have shown limited predictive accuracy in identifying patients at high risk of recurrence [14]. The inability to reliably predict recurrence not only hampers patient selection and counseling but also complicates post-procedural management and follow-up strategies [15]. In recent years, ML methods have demonstrated significant potential in clinical prediction modeling, particularly for complex cardiovascular conditions [16–21]. By integrating large-scale, multidimensional data and capturing non-linear interactions among variables, ML algorithms can outperform traditional statistical methods in risk stratification [22, 23]. Several studies have explored ML-based approaches for predicting AF ablation outcomes [24–26]. However, most have been limited by small sample sizes, heterogeneous data quality, and insufficient validation [27]. Importantly, evidence derived from real-world, single-center cohorts remains scarce, yet such data may offer meaningful insights into the practical applicability of these models in clinical settings.

While previous studies have compared multiple ML algorithms for predicting AF recurrence, there remains a need for models that are not only accurate but also interpretable, clinically applicable, and rigorously validated in real-world settings. Therefore, in this study, we aimed not merely to compare algorithms, but to develop, validate, and interpret a clinically useful prediction tool for AF recurrence after catheter ablation using a comprehensive single-center dataset. Our objective was to integrate a wide array of clinical and laboratory variables into an advanced predictive framework, with an emphasis on model interpretability and clinical utility. By providing an individualized, explainable recurrence risk estimation, the proposed model may assist clinicians in refining patient selection, tailoring post-ablation follow-up strategies, and ultimately improving long-term outcomes in patients with AF.

## Methods

### Study design and data source

This study was conducted to develop and validate a risk prediction model for AF recurrence following catheter ablation. We consecutively enrolled patients who underwent first-time AF ablation at the Provincial Hospital Affiliated to Fuzhou University between August 31, 2016, and August 31, 2023. Data were extracted from electronic medical records (EMRs), encompassing demographic characteristics, clinical history, lifestyle factors, laboratory parameters, and post-ablation follow-up records. The study protocol was approved by the Institutional Ethics Committee and adhered to the Declaration of Helsinki. The reporting follows the STROBE and TRIPOD guidelines. Because of the retrospective design, the requirement for informed consent was waived and all data were anonymized before analysis.

### Study participants

Inclusion criteria were: (1) ECG- or Holter-confirmed AF (paroxysmal, persistent, or permanent); (2) age ≥ 18 years; (3) undergoing initial radiofrequency or cryoballoon ablation; (4) availability of complete clinical information; and (5) completed follow-up of ≥ 3 months post-ablation. Exclusion criteria were: (1) missing key variables; (2) comorbid malignancies or severe organ failure; (3) lost to follow-up within 3 months; or (4) AF recurrence attributed to procedural complications such as pericardial effusion rather than arrhythmia itself.

### Variables and outcome definition

The primary outcome was AF recurrence after catheter ablation, defined as any documented episode of symptomatic or asymptomatic AF, atrial flutter, or atrial tachycardia lasting ≥ 30 s, confirmed by electrocardiogram or Holter monitoring occurring at least 3 months after the procedure to exclude early transient arrhythmias. Recurrence was coded as a binary variable (“Yes” or “No”). Predictor variables covered demographic characteristics, lifestyle factors, clinical history, cardiac parameters, medication use, laboratory measures, and risk scores. Demographic variables included age (years), sex (categorized as Female or Male), body mass index (BMI, kg/m²), height (cm) and weight (kg). Lifestyle factors included smoking and alcohol consumption (both categorized as No or Yes). Clinical history variables comprised: prior pacemaker (No/Yes), history of stroke (No/Yes), transient ischemic attack (TIA; No/Yes), coronary heart disease (CHD; No/Yes), coronary stent surgery (No/Yes), hypertension (No/Yes), diabetes (No/Yes), hyperlipidemia (No/Yes), hypertrophic cardiomyopathy (HCM; No/Yes), and heart failure (HF; No/Yes). Cardiac structural and functional parameters included left atrial area (LAA, cm²), left ventricular area (LVA, cm²), and ejection fraction (EF, %). Medication use variables included postoperative arrhythmia medication (categorized as No, amiodarone, or beta-receptor antagonist), preoperative use of amiodarone (No/Yes), preoperative use of anticoagulant drugs (No/Yes), and preoperative use of antiplatelet drugs (No/Yes). Laboratory measures encompassed biomarkers of cardiac function, inflammation, metabolism, coagulation, and organ function, including: B-type natriuretic peptide (BNP, pg/mL), neutrophil count (×10⁹/L), leucocyte count (×10⁹/L), platelet count (×10⁹/L), alanine aminotransferase (ALT, U/L), hemoglobin (g/L), apolipoprotein A1 (ApoA1, g/L), apolipoprotein B (ApoB, g/L), albumin (g/L), aspartate aminotransferase (AST, U/L), blood urea nitrogen (BUN, mmol/L), total cholesterol (TCHOL, mmol/L), creatine kinase (CK, U/L), alkaline phosphatase (ALP, U/L), creatine kinase-MB (CK-MB, ng/mL), creatinine (Cr, µmol/L), lactate dehydrogenase (LDH, U/L), calcium (Ca, mmol/L), direct bilirubin (DBil, µmol/L), globulin (g/L), hypoglycemia indicator, high-density lipoprotein (HDL, mmol/L), gamma-glutamyl transferase (GGT, U/L), sodium (Na, mmol/L), potassium (K, mmol/L), triglyceride (mmol/L), uric acid (UA, µmol/L), fibrin degradation product (FDP, µg/mL; normal < 5 µg/mL), low-density lipoprotein (LDL, mmol/L), total bilirubin (TBil, µmol/L), antithrombin III (AT-III, %), international normalized ratio (INR), D-dimer (D-D, mg/L; normal < 0.5 µg/mL), fibrinogen (FIB, g/L), thrombin time (TT, s), activated partial thromboplastin time (APTT, s) and prothrombin time (PT, s). Risk scores included the CHA₂DS₂-VASc and HAS-BLED scores, both treated as continuous variables based on computed values. All continuous variables were assessed using standard laboratory assays or clinical evaluations.

### Data preprocessing

The data were all processed using R software. Missing data were handled by listwise deletion using the na.omit() function, as the overall missing rate was low (< 1.5%) and confirmed to be missing completely at random (MCAR) via Little’s test. While multiple imputation is a more robust alternative, its use was deemed unnecessary given the minimal missing data. Categorical variables (such as sex, smoking) were encoded as factor variables with descriptive labels, while continuous variables (such as ALP, FDP) were retained in their original scale without normalization. The dataset was randomly divided into a training set (60%) and a validation set (40%) using the createDataPartition function to ensure balanced outcome distribution between groups.

### Variable selection

To enhance model generalizability, prevent overfitting, and improve clinical translatability by identifying a parsimonious set of predictors, a two-step variable screening method was employed. This two-step variable selection was employed to reduce dimensionality and mitigate overfitting, given the moderate sample size relative to the number of predictors. While embedded feature selection within ML models is an alternative, our approach aimed to enhance model interpretability and stability. Firstly, in the initial screening, variables with clinical relevance (based on AF recurrence literature) and no collinearity (variance inflation factor < 5) are retained. Secondly, in the statistical screening, the stepwise regression method is adopted to screen the variables, and univariate logistic regression (through the autoReg package) is used to retain the variables with a P value < 0.05. Then, the variables with P value < 0.05 in the univariate logistic regression were included in the multivariate logistic regression analysis. Finally, only the variables with a P value < 0.05 in the multivariate logistic regression were selected as the final predictor set for the subsequent development of all machine learning models. This rigorous process ensured that the models were built on a concise and robust subset of features.

### Model development and hyperparameter optimization

In the training set, prediction models were constructed using eleven machine learning algorithms: Naive Bayes (NB), Classification and Regression Trees (CART), Logistic Regression (LR), Random Forest (RF), Linear Discriminant Analysis (LDA), Support Vector Machine (SVM), K-Nearest Neighbors (KNN), Neural Network (NNET), Gradient Boosting Machine (GBM), XGBoost and Elastic Net (GLMNET). Internal training was conducted via 10-fold cross-validation (method = “cv”, number = 10), and bootstrap validation (method = “boot”, number = 200) was employed to evaluate model robustness. The bootstrap validation was performed within the training set using 200 resamples, independent of the 10-fold cross-validation used for hyperparameter tuning, to assess the model’s internal stability. The caret package was used for model training, with the AUC serving as the primary metric for both hyperparameter tuning and model selection. Hyperparameter settings were as follows: GLM and LDA used default parameters; RF tuned mtry (3–8) and min.node.size (10, 20), with fixed num.trees = 1000 and splitrule = “gini”; SVM automatically selected sigma and tuned C (1–10); KNN adjusted k (5–15); NNET tuned size (3–7) and decay (0.001–0.1); GBM adjusted n.trees (500–1000) and interaction.depth (3–5); CART used cp. = 0.001; XGBoost tuned nrounds (100–500) and max_depth (3–7) with eta = 0.1; NB applied Laplace smoothing (fL = 0.01); and GLMNET tuned alpha (0–1) with lambda selected automatically through cross-validation. The model demonstrating the highest AUC in the training set, along with consistent performance in the validation set, was selected as optimal. Performance was assessed in both training set and validation set across three aspects: discrimination, calibration, and clinical utility. For discrimination, the AUC was computed using the pROC package, reported with 95% confidence intervals (95% CI). Classification metrics—including F1 score, specificity, sensitivity and positive predictive value (PPV)—were derived from confusion matrices (using the confusionMatrix function) at a probability threshold of 0.5. PR curves and the area under these curves (PR-AUC) were calculated via the PRROC package to evaluate performance on imbalanced data. Calibration was assessed using calibration curves with 10 bins, generated with the calibration function and a loess-smoothed line to compare predicted and observed recurrence probabilities. Brier scores are calculated based on the mean square deviation between the predicted probabilities and the actual labels and are used to quantify the calibration error (range 0–1, lower values indicating better calibration). Clinical utility was evaluated using DCA implemented in the decisionCurve package, which quantified net clinical benefit across threshold probabilities from 0 to 1. A model was deemed clinically useful if its net benefit surpassed both the “treat all” and “treat none” strategies. Additionally, a nomogram based on LR model was developed to provide an intuitive visualization of the predictive model. To improve interpretability, SHAP values were visualized using beeswarm plots to illustrate the contribution of each predictor to the estimated risk of recurrence.

### Statistical software

All statistical analyses were performed using R software (version 4.5.0; https://www.r-project.org/). Continuous variables were presented as mean ± standard deviation or median with interquartile range (IQR), based on their distribution normality assessed by the Shapiro-Wilk test. Categorical variables were summarized as frequencies and percentages. Differences between groups were examined using independent samples t-test or Mann-Whitney U test for continuous variables, and Chi-square or Fisher’s exact test for categorical variables, as appropriate. Key R packages employed in this study included: readxl, dplyr, and caret for data management and preprocessing; AutoReg for automated variable selection; ranger, xgboost, nnet, and glmnet for model training and implementation; pROC, PRROC, and broom for model performance evaluation and metrics extraction; ggplot2, shapviz, and rms for result visualization and nomogram construction. In the logistic regression model, associations between predictors and the outcome were quantified using odds ratios (OR) with 95% confidence intervals (CI). A two-sided P-value < 0.05 was considered statistically significant for all analyses.

## Results

### Baseline information

As shown in Table 1, of the 438 patients with AF included in this study who underwent catheter ablation, 147 (33.6%) experienced recurrence during follow-up. The median age of the total population was 63 (57, 69) years, with 64 (57, 70) years in the non-recurrence group and 63 (56, 69) years in the recurrence group, and the difference between the two groups was not statistically significant (*P* = 0.303). Multiple baseline characteristics demonstrated statistically significant differences (*P* < 0.05) between the recurrence and non-recurrence groups. Demographic factors included a higher proportion of males in the recurrence group (59.86% vs. 47.42%, *P* = 0.018). Lifestyle factors significantly differed, with a lower prevalence of smoking (12.24% vs. 23.37%, *P* = 0.008) and a higher prevalence of alcohol consumption (53.74% vs. 42.61%, *P* = 0.035) in the recurrence group. The recurrence group also had a higher proportion of individuals without a history of pacemaker implantation (59.86% vs. 46.05%, *P* = 0.009). Comorbidity profiles were markedly different. The recurrence group had a lower prevalence of stroke (40.82% vs. 51.55%, *P* = 0.043), transient ischemic attack (TIA) (16.33% vs. 27.84%, *P* = 0.011), coronary heart disease (CHD) (40.82% vs. 54.30%, *P* = 0.010), hypertension (44.90% vs. 56.36%, *P* = 0.030), and peripheral artery disease (PAD) (44.90% vs. 57.04%, *P* = 0.021). Conversely, the recurrence group had a higher prevalence of hyperlipidemia (58.50% vs. 47.08%, *P* = 0.031), heart failure (HF) (50.34% vs. 39.52%, *P* = 0.040), and preoperative use of amiodarone (57.82% vs. 46.05%, *P* = 0.026). Hypertrophic cardiomyopathy (HCM) was also more common in the recurrence group (5.44% vs. 1.03%, *P* = 0.008). Cardiac structural parameters were significantly worse in the recurrence group, including a larger left atrial area (LAA) (median 4.06 vs. 3.80 cm, *P* = 0.005) and a larger left ventricular area (LVA) (median 4.65 vs. 4.59 cm, *P* = 0.015). Laboratory findings further distinguished the groups. The recurrence group had higher levels of B-type natriuretic peptide (BNP) (median 545.83 vs. 334.82 pg/mL, *P* = 0.007), creatine kinase-MB (CK-MB) (median 14.04 vs. 12.53 ng/mL, *P* = 0.005), alkaline phosphatase (ALP) (median 73.67 vs. 65.59 U/L, *P* = 0.044), sodium (Na) (median 143.43 vs. 142.51 mmol/L, *P* = 0.044), and fibrin degradation product (FDP) (median 2.09 vs. 1.72 µg/mL, *P* = 0.010). They also had lower levels of high-density lipoprotein (HDL) (median 1.13 vs. 1.24 mmol/L, *P* = 0.027) and D-dimer (D-D) (median 0.14 vs. 0.15 mg/L, *P* = 0.036).

**Table 1:** Baseline information on the study population

Variable	Total (*n* = 438)	No (*n* = 291)	Yes (*n* = 147)	*p*-value
Age, Median (Q1,Q3)	63 (57, 69)	64 (57, 70)	63 (56, 69)	0.303
Sex, n (%)				0.018
Female	212 (48.4)	153 (52.58)	59 (40.14)	
Male	226 (51.6)	138 (47.42)	88 (59.86)	
LAA, Median (Q1,Q3)	3.86 (3.45, 4.43)	3.8 (3.41, 4.32)	4.06 (3.52, 4.68)	0.005
LVA, Median (Q1,Q3)	4.61 (4.31, 4.86)	4.59 (4.3, 4.84)	4.65 (4.4, 5.07)	0.015
EF, Median (Q1,Q3)	58.13 (48.76, 67.05)	59.14 (49.65, 67.32)	57.3 (47.29, 66.34)	0.191
Height, Median (Q1,Q3)	166 (160, 172)	166 (160, 172)	165 (160, 172)	0.635
Weight, Median (Q1,Q3)	69.48 (55.75, 83.43)	70.34 (56.78, 84.82)	67.32 (54.95, 80.91)	0.343
BMI, Median (Q1,Q3)	24.02 (20.51, 27.78)	24.04 (20.74, 27.77)	23.92 (20.24, 27.66)	0.55
Smoking, n (%)				0.008
No	352 (80.37)	223 (76.63)	129 (87.76)	
Yes	86 (19.63)	68 (23.37)	18 (12.24)	
Drinking, n (%)				0.035
No	235 (53.65)	167 (57.39)	68 (46.26)	
Yes	203 (46.35)	124 (42.61)	79 (53.74)	
PPO, n (%)				0.009
No	222 (50.68)	134 (46.05)	88 (59.86)	
Yes	216 (49.32)	157 (53.95)	59 (40.14)	
History, Median (Q1,Q3)	66.49 (29.26, 124.69)	69.17 (31.36, 126.04)	58.18 (27.18, 113.86)	0.279
Stroke, n (%)				0.043
No	228 (52.05)	141 (48.45)	87 (59.18)	
Yes	210 (47.95)	150 (51.55)	60 (40.82)	
TI, n (%)				0.011
No	333 (76.03)	210 (72.16)	123 (83.67)	
Yes	105 (23.97)	81 (27.84)	24 (16.33)	
CHD, n (%)				0.01
No	220 (50.23)	133 (45.7)	87 (59.18)	
Yes	218 (49.77)	158 (54.3)	60 (40.82)	
CSS, n (%)				0.031
No	247 (56.39)	153 (52.58)	94 (63.95)	
Yes	191 (43.61)	138 (47.42)	53 (36.05)	
Hypertension, n (%)				0.03
No	208 (47.49)	127 (43.64)	81 (55.1)	
Yes	230 (52.51)	164 (56.36)	66 (44.9)	
Diabetes, Median (Q1,Q3)	0.33 (0.17, 0.59)	0.33 (0.18, 0.59)	0.33 (0.16, 0.6)	0.823
HC, n (%)				0.008
No	427 (97.49)	288 (98.97)	139 (94.56)	
Yes	11 (2.51)	3 (1.03)	8 (5.44)	
PAM, n (%)				0.099
No	212 (48.4)	151 (51.89)	61 (41.5)	
Amiodarone	115 (26.26)	69 (23.71)	46 (31.29)	
Beta-receptor antagonist	111 (25.34)	71 (24.4)	40 (27.21)	
CHA₂DS₂-VASc, Median (Q1,Q3)	2.36 (1, 3.97)	2.36 (0.92, 3.97)	2.37 (1.12, 3.94)	0.979
HAS-BLED, Median (Q1,Q3)	1.27 (0.56, 2.12)	1.28 (0.58, 2.11)	1.17 (0.53, 2.12)	0.505
HF, n (%)				0.04
No	249 (56.85)	176 (60.48)	73 (49.66)	
Yes	189 (43.15)	115 (39.52)	74 (50.34)	
PA, n (%)				0.026
No	219 (50)	157 (53.95)	62 (42.18)	
Yes	219 (50)	134 (46.05)	85 (57.82)	
PAD, n (%)				0.021
No	206 (47.03)	125 (42.96)	81 (55.1)	
Yes	232 (52.97)	166 (57.04)	66 (44.9)	
PAND, n (%)				0.034
No	383 (87.44)	247 (84.88)	136 (92.52)	
Yes	55 (12.56)	44 (15.12)	11 (7.48)	
Hyperlipidemia, n (%)				0.031
No	215 (49.09)	154 (52.92)	61 (41.5)	
Yes	223 (50.91)	137 (47.08)	86 (58.5)	
BNP, Median (Q1,Q3)	387.56 (176.11, 781.54)	334.82 (151.2, 719.79)	545.83 (225.28, 870.77)	0.007
Leucocyte, Median (Q1,Q3)	6.58 (4.56, 8.41)	6.57 (4.48, 8.37)	6.67 (4.8, 8.5)	0.561
Neutrophil, Median (Q1,Q3)	3.86 (2.3, 5.48)	3.89 (2.13, 5.69)	3.82 (2.41, 5.19)	0.597
Hemoglobin, Median (Q1,Q3)	139.85 (123.92, 156.98)	137.94 (122.54, 155.25)	144.09 (128.69, 158.9)	0.04
Platelet, Median (Q1,Q3)	225.01 (150.01, 321.21)	223.09 (142.12, 317.59)	237.35 (151.09, 336.18)	0.43
ALP, Median (Q1,Q3)	68.37 (50.88, 89.04)	65.59 (50.47, 84.32)	73.67 (54.31, 93.54)	0.044
ALT, Median (Q1,Q3)	30.6 (17.79, 51.03)	31.63 (18.24, 52.29)	28.65 (16.72, 49.26)	0.184
AST, Median (Q1,Q3)	24.63 (16.16, 34.73)	24.52 (15.98, 35.78)	24.76 (17, 33.52)	0.956
ApoA1, Median (Q1,Q3)	1.35 (1.08, 1.62)	1.36 (1.1, 1.62)	1.28 (1.05, 1.61)	0.28
ApoB, Median (Q1,Q3)	0.94 (0.64, 1.32)	0.94 (0.63, 1.34)	0.96 (0.64, 1.32)	0.918
Albumin, Median (Q1,Q3)	42.4 (35.73, 47.6)	41.81 (35.04, 47.32)	43.26 (36.46, 48.03)	0.419
BUN, Median (Q1,Q3)	5.53 (4.4, 7.11)	5.44 (4.3, 6.83)	5.91 (4.53, 7.62)	0.05
TCHOL, Median (Q1,Q3)	4.34 (3.23, 5.6)	4.34 (3.21, 5.43)	4.34 (3.27, 5.69)	0.359
CK, Median (Q1,Q3)	100.2 (56.21, 163.91)	97.12 (55.16, 166.36)	104.2 (62.06, 159.46)	0.914
CKMB, Median (Q1,Q3)	13.04 (9.86, 16.77)	12.53 (9.09, 16.25)	14.04 (10.95, 17.25)	0.005
Cr, Median (Q1,Q3)	78.04 (58.79, 106.93)	77.35 (59.2, 107.56)	79.23 (57.73, 106.12)	0.803
Ca, Median (Q1,Q3)	2.37 (2.19, 2.64)	2.39 (2.2, 2.65)	2.34 (2.19, 2.61)	0.464
DBB, Median (Q1,Q3)	5.61 (3.67, 8.52)	5.7 (3.71, 8.81)	5.34 (3.63, 7.94)	0.239
GGT, Median (Q1,Q3)	47.52 (25.46, 78.01)	44.93 (25.4, 78.87)	51.63 (25.82, 70.85)	0.902
Globulin, Median (Q1,Q3)	27.17 (22.21, 31.59)	27.36 (22.26, 31.55)	26.95 (22.14, 31.69)	0.496
Hypoglycemia, Median (Q1,Q3)	5.96 (4.74, 7.54)	5.96 (4.71, 7.48)	5.96 (4.83, 7.65)	0.622
HDL, Median (Q1,Q3)	1.21 (0.9, 1.5)	1.24 (0.96, 1.54)	1.13 (0.83, 1.43)	0.027
K, Median (Q1,Q3)	4.2 (3.77, 4.72)	4.23 (3.77, 4.71)	4.16 (3.79, 4.74)	0.969
LDH, Median (Q1,Q3)	177.82 (139.75, 225.02)	179.4 (138.35, 234.01)	175.81 (143.18, 215.26)	0.509
LDL, Median (Q1,Q3)	2.71 (1.85, 4.04)	2.73 (1.8, 3.99)	2.68 (1.89, 4.1)	0.83
Na, Median (Q1,Q3)	142.74 (140.53, 144.75)	142.51 (140.43, 144.47)	143.43 (140.64, 145.11)	0.044
TOB, Median (Q1,Q3)	11.49 (7.23, 17.62)	10.95 (6.86, 16.46)	12.94 (8.18, 18.37)	0.026
Triglyceride, Median (Q1,Q3)	2.3 (1.35, 3.6)	2.24 (1.3, 3.63)	2.35 (1.47, 3.54)	0.911
UA, Median (Q1,Q3)	373.07 (240.78, 494.41)	378.5 (232.37, 504.58)	365.75 (259.97, 477.88)	0.797
APTT, Median (Q1,Q3)	32.33 (24.29, 44.95)	32.07 (24.53, 43.46)	34.48 (23.72, 46.1)	0.594
AntithrombinIII, Median (Q1,Q3)	86.07 (69.89, 103.39)	86.52 (72.08, 104.78)	84.59 (67.34, 101.73)	0.432
DD, Median (Q1,Q3)	0.14 (0.09, 0.25)	0.15 (0.09, 0.27)	0.14 (0.08, 0.21)	0.036
FDP, Median (Q1,Q3)	1.86 (1.09, 2.96)	1.72 (1.01, 2.89)	2.09 (1.32, 3.2)	0.01
FIB, Median (Q1,Q3)	2.89 (2.05, 4.01)	3.01 (2.09, 4.28)	2.8 (2, 3.62)	0.088
INR, Median (Q1,Q3)	1.61 (1.19, 2.36)	1.63 (1.18, 2.38)	1.59 (1.21, 2.33)	0.858
PT, Median (Q1,Q3)	16.38 (11.89, 21.79)	16.65 (11.7, 22.38)	16.1 (12.66, 21.01)	0.79
TT, Median (Q1,Q3)	38.14 (19.55, 59.69)	36.54 (18.74, 57.08)	41.08 (24.27, 62.11)	0.058

*ALT* Alanine aminotransferase, ApoA1 Apolipoprotein A, *BMI* Body mass index, *BNP*B-type natriuretic peptide, *BUN* Blood urea nitrogen, *Ca* Calcium, *CHD* Coronary heart disease, *CK* Creatine kinase, *CK-MB* Creatine kinase-MB, *Cr* creatinine, *ALP* Alkaline phosphatase, *CSS *Coronary stent surgery, *GGT* Gamma-glutamyl transferase, *DBB* Direct bilirubin, *DD* D-dimer, *EF* Ejection fraction, *ApoB* Apolipoprotein B, *FDP* Fibrin degradation product, *FIB* Fibrinogen, *HDL* High-density lipoprotein, *AST* Aspartate aminotransferase, *HF* Heart failure, *HC* Hypertrophic cardiomyopathy, *INR* International normalized ratio, *LAA* Left atrial area, *LDH* Lactate dehydrogenase, *LDL* Low-density lipoprotein, *LVA* Left ventricular area, *PA* Preoperative use of amiodarone, *PAD* Preoperative use of anticoagulant drugs, *PAM* Postoperative arrhythmia medication, *PAND* Preoperative use of antiplatelet drugs, *PPO* Post–pacemaker operation, *PT* Prothrombin time, *TCHOL* Total cholesterol, *TI* Transient ischemia, *TOB* Total bilirubin, *TT* Thrombin time, *UA* Uric acid

### Univariate and multivariate logistic regression analysis

In the univariate logistic regression analysis, multiple variables demonstrated significant associations with AF recurrence (*P* < 0.05), including alcohol consumption, sex, smoking, history of pacemaker implantation, transient ischemic attack (TIA), coronary heart disease (CHD), hypertension, hyperlipidemia, hypertrophic cardiomyopathy (HCM), preoperative use of amiodarone, preoperative use of anticoagulant drugs, hemoglobin, alkaline phosphatase (ALP), D-dimer (D-D), fibrin degradation product (FDP), and total bilirubin (TBil). These variables were subsequently included in the multivariate logistic regression model. It is important to note that the associations reported below from the multivariate logistic regression analysis represent statistical adjustments for the included covariates. The direction and magnitude of these associations should be interpreted with caution, considering clinical plausibility, as they may be influenced by unmeasured confounding, collinearity, or specific characteristics of the study cohort. After adjusting for potential confounders in the multivariate analysis, alcohol consumption (OR = 2.12, 95% CI: 1.15–3.91, *P* = 0.017), preoperative use of amiodarone (OR = 2.03, 95% CI: 1.12–3.66, *P* = 0.019), and postoperative use of beta-receptor antagonists (OR = 2.38, 95% CI: 1.11–5.10, *P* = 0.026) were significantly associated with an increased risk of AF recurrence. Conversely, smoking (OR = 0.38, 95% CI: 0.15–0.95, *P* = 0.038), hypertension (OR = 0.47, 95% CI: 0.26–0.85, *P* = 0.012), and peripheral artery disease (PAD) (OR = 0.43, 95% CI: 0.22–0.82, *P* = 0.011) were associated with a decreased risk of recurrence. Additionally, elevated FDP levels were associated with increased recurrence risk (OR = 1.22, 95% CI: 1.02–1.46, *P* = 0.027). The variables identified as statistically significant in this multivariate analysis constituted the refined predictor set used for training and comparing the eleven machine learning models, substantially reducing the dimensionality from the initial set of candidate variables. The results of the logistic regression analysis are shown in Table 2.

**Table 2:** Univariate and multivariate logistic regression analyses of factors associated with recurrence

Character	Univariate Analysis OR (95% CI)	*P* value	Multivariate Analysis OR (95% CI)	*P* value
Age (years)	0.98 (0.95–1.01)	0.183	/	/
LAA (cm)	1.39 (0.94–2.05)	0.095	/	/
LVA (cm)	1.43 (0.88–2.31)	0.144	/	/
EF (%)	0.99 (0.97–1.01)	0.297	/	/
Height (cm)	1.01 (0.97–1.04)	0.731	/	/
Weight (kg)	0.99 (0.97–1.01)	0.227	/	/
BMI (kg/m²)	0.99 (0.94–1.05)	0.810	/	/
History (months)	1.00 (0.99–1.00)	0.173	/	/
Diabetes	0.98 (0.49–1.97)	0.955	/	/
CHA₂DS₂-VASc score	1.00 (0.83–1.20)	0.993	/	/
HAS-BLED score	0.76 (0.56–1.04)	0.085	/	/
BNP (pg/mL)	1.00 (1.00–1.00)	0.391	/	/
Leucocyte (×10⁹/L)	1.10 (0.99–1.23)	0.081	/	/
Neutrophil (×10⁹/L)	0.95 (0.83–1.08)	0.411	/	/
Hemoglobin (g/L)	1.01 (1.00–1.03)	0.027	/	/
Platelet (×10⁹/L)	1.00 (1.00–1.00)	0.284	/	/
ALP (U/L)	1.01 (1.00–1.02)	0.105	/	/
ALT (U/L)	1.00 (0.98–1.01)	0.395	/	/
AST (U/L)	0.99 (0.97–1.02)	0.572	/	/
ApoA1 (g/L)	0.68 (0.31–1.45)	0.316	/	/
ApoB (g/L)	1.32 (0.69–2.54)	0.403	/	/
Albumin (g/L)	1.01 (0.98–1.05)	0.542	/	/
BUN (mmol/L)	1.07 (0.93–1.24)	0.328	/	/
TCHOL (mmol/L)	1.03 (0.86–1.24)	0.727	/	/
CK (U/L)	1.00 (0.99–1.00)	0.329	/	/
CKMB (ng/mL)	1.01 (0.97–1.05)	0.712	/	/
Cr (µmol/L)	1.00 (0.99–1.01)	0.633	/	/
Ca (mmol/L)	0.75 (0.36–1.55)	0.432	/	/
DBB	0.98 (0.90–1.06)	0.604	/	/
GGT (U/L)	1.00 (0.99–1.01)	0.759	/	/
Globulin (g/L)	1.02 (0.97–1.07)	0.386	/	/
Glycemia	1.01 (0.88–1.16)	0.846	/	/
HDL (mmol/L)	0.74 (0.43–1.27)	0.270	/	/
K (mmol/L)	1.23 (0.79–1.90)	0.354	/	/
LDH (U/L)	1.00 (0.99–1.00)	0.571	/	/
LDL (mmol/L)	1.05 (0.86–1.27)	0.651	/	/
Na (mmol/L)	1.01 (0.92–1.11)	0.807	/	/
TOB (µmol/L)	1.04 (1.00–1.08)	0.034	1.03 (0.98–1.07)	0.217
Triglyceride (mmol/L)	1.08 (0.92–1.26)	0.368	/	/
UA (µmol/L)	1.00 (1.00–1.00)	0.787	/	/
APTT (s)	1.00 (0.98–1.02)	0.931	/	/
Antithrombin III (%)	1.00 (0.98–1.01)	0.448	/	/
DD (mg/L)	0.58 (0.25–1.32)	0.195	/	/
FDP (µg/mL)	1.17 (1.00–1.37)	0.045	1.22 (1.02–1.46)	0.027
FIB (g/L)	0.85 (0.68–1.07)	0.175	/	/
INR	0.95 (0.72–1.26)	0.716	/	/
PT (s)	0.98 (0.94–1.02)	0.303	/	/
TT (s)	1.00 (0.99–1.01)	0.433	/	/
Hyperlipidemia	—	/	—	/
No	1.00 (reference)	/	/	/
Yes	1.67 (1.00–2.80)	0.052	/	/
Drinking		/	/	/
No	1.00 (reference)	/	1.00 (reference)	/
Yes	2.01 (1.20–3.38)	0.008	2.12 (1.15–3.91)	0.017
Sex	—	/	/	/
Female	1.00 (reference)	/	1.00 (reference)	/
Male	1.67 (1.00–2.81)	0.052	1.32 (0.68–2.54)	/
Smoking	—	/	—	/
No	1.00 (reference)	/	1.00 (reference)	/
Yes	0.58 (0.29–1.15)	0.117	0.38 (0.15–0.95)	0.038
PPO	—	/	—	/
No	1.00 (reference)	/	1.00 (reference)	/
Yes	0.57 (0.34–0.96)	0.034	0.56 (0.31–1.01)	0.054
TI	—	/	—	/
No	1.00 (reference)	/	1.00 (reference)	/
Yes	0.42 (0.22–0.80)	0.009	0.50 (0.24–1.04)	0.062
Stroke	—	/	—	/
No	1.00 (reference)	/	/	/
Yes	0.79 (0.47–1.32)	0.364	/	/
CHD	—	/	—	/
No	1.00 (reference)	/	1.00 (reference)	/
Yes	0.55 (0.33–0.92)	0.022	0.50 (0.28–0.89)	0.019
CSS	—	/	—	/
No	1.00 (reference)	/	/	/
Yes	0.73 (0.44–1.24)	0.245	/	/
Hypertension	—	/	—	/
No	1.00 (reference)	/	1.00 (reference)	/
Yes	0.52 (0.31–0.87)	0.014	0.47 (0.26–0.85)	0.012
HF	—	/	—	/
No	1.00 (reference)	/	/	/
Yes	1.19 (0.71–1.98)	0.506	/	/
HC	—	/	—	/
No	1.00 (reference)	/	1.00 (reference)	/
Yes	4.14 (1.01–16.98)	0.048	5.40 (1.02–28.48)	0.047
PA	—	/	—	/
No	1.00 (reference)	/	1.00 (reference)	/
Yes	1.84 (1.09–3.10)	0.022	2.03 (1.12–3.67)	0.019
PAD	—	/	—	/
No	1.00 (reference)	/	1.00 (reference)	/
Yes	0.59 (0.35–0.98)	0.042	0.56 (0.31-1.00)	0.051
PAND	—	/	/	/
No	1.00 (reference)	/	/	/
Yes	0.43 (0.18–1.02)	0.057	/	/
PAM	—	/	—	/
No	1.00 (reference)	/	1.00 (reference)	/
Amiodarone	1.74 (0.92–3.30)	0.088	1.52 (0.74–3.15)	0.256
Beta-receptor antagonist	2.54 (1.37–4.70)	0.003	2.33 (1.16–4.68)	0.018

*ALT* Alanine aminotransferase, *ApoA1* Apolipoprotein A1, *BMI* Body mass index, *BNP* B-type natriuretic peptide, *BUN* Blood urea nitrogen, *Ca* Calcium, *CHD* Coronary heart disease, *CK* Creatine kinase, *CK-MB* Creatine kinase-MB, *Cr* Creatinine, *ALP* Alkaline phosphatase, *CSS* Coronary stent surgery, *GGT* Gamma-glutamyl transferase, *DBB* Direct bilirubin, *DD* D-dimer, *EF* Ejection fraction, *ApoB* Apolipoprotein B, *FDP* Fibrin degradation product, *FIB* Fibrinogen, *HDL* High-density lipoprotein, *AST* Aspartate aminotransferase, *HF* Heart failure, *HC* Hypertrophic cardiomyopathy, *INR* International normalized ratio, *LAA* Left atrial area, *LDH* Lactate dehydrogenase, *LDL* Low-density lipoprotein, *LVA* Left ventricular area, *PA* Preoperative use of amiodarone, *PAD* Preoperative use of anticoagulant drugs, *PAM* Postoperative arrhythmia medication, *PAND* Preoperative use of antiplatelet drugs, *PPO* Post–pacemaker operation, *PT* Prothrombin time, *TCHOL* Total cholesterol, *TI *Transient ischemia, *TOB* Total bilirubin. *TT* Thrombin time, *UA* Uric acid

### Development and validation of 11 ML models

**Fig. 1:**
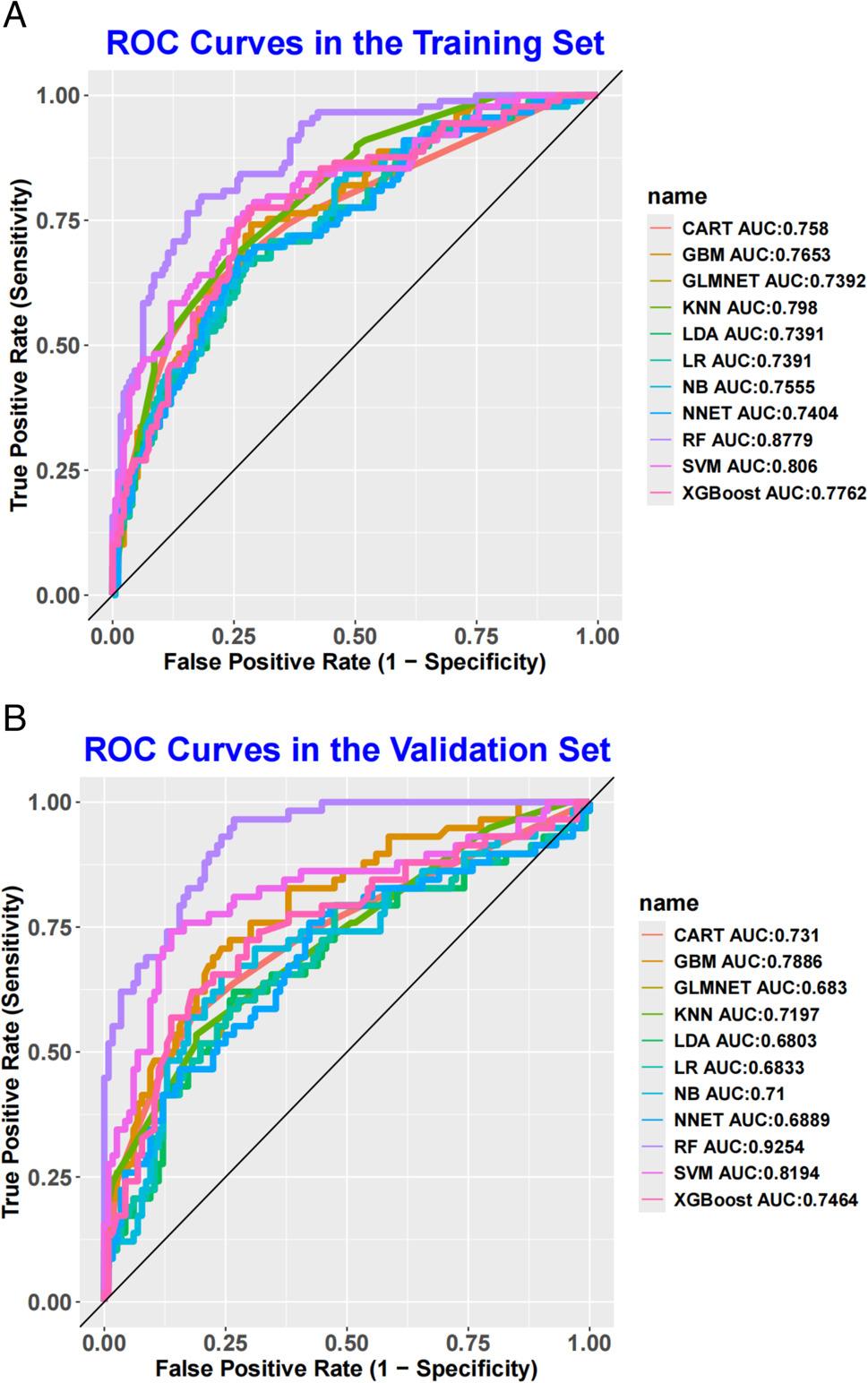
ROC curve analysis of eleven ML algorithms in the training set (A) and validation set (B)

**Fig. 2:**
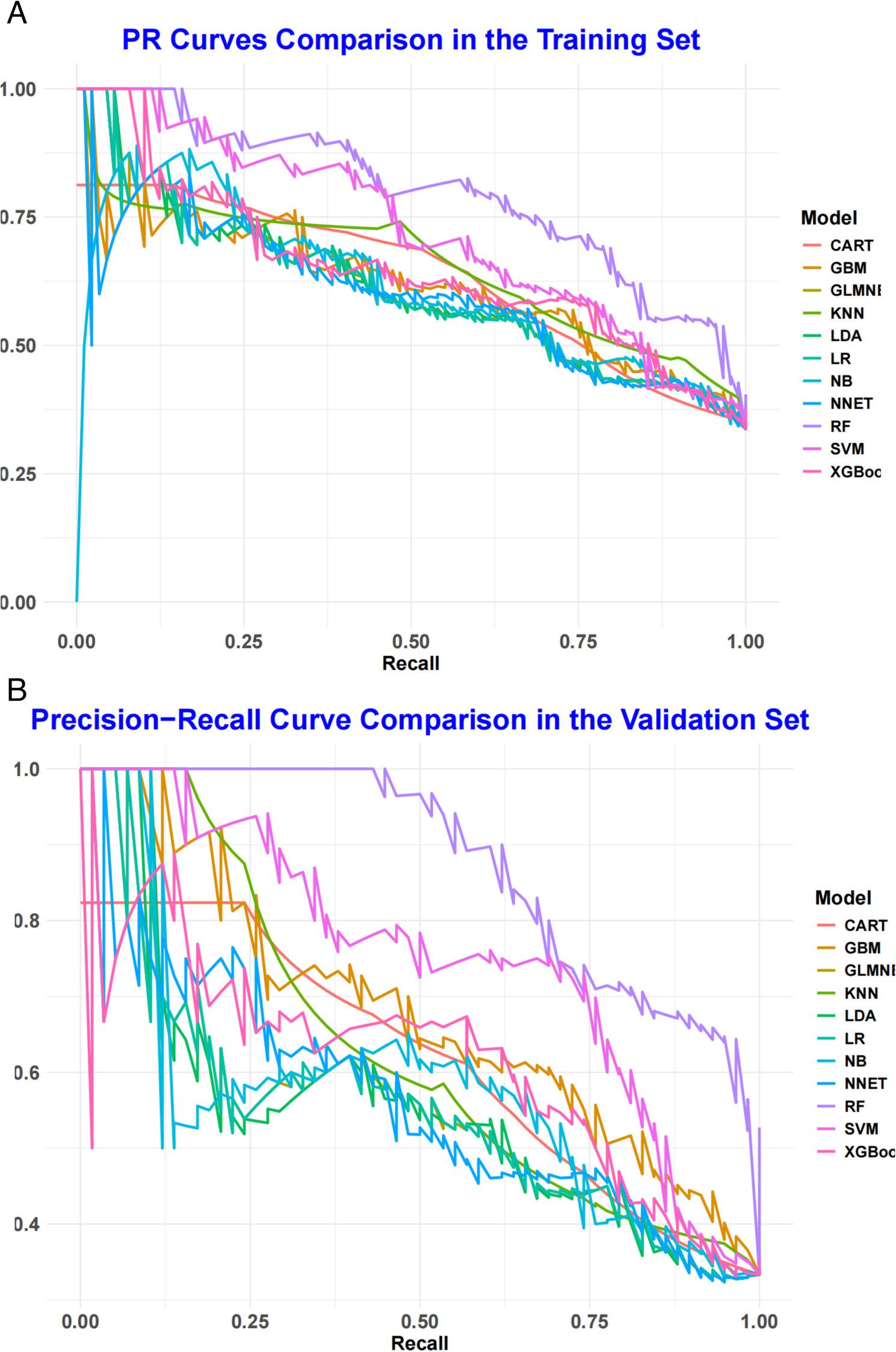
PR curve analysis of eleven ML algorithms in the training set (A) and validation set (B)

To evaluate and validate the performance of 11 ML models in predicting AF recurrence after catheter ablation, we first constructed ROC curves to assess discriminatory performance. In the training set (Fig. 1A), the RF model achieved the highest AUC of 0.878, followed by SVM (AUC = 0.806), XGBoost (0.776), CART (0.758), NB (0.756), GBM (0.765), NNET (0.740), GLMNET (0.739), LDA (0.739), LR (0.739), and KNN (0.798). In the validation set (Fig. 1B), the RF model again demonstrated the best performance among all models, with an AUC of 0.925—representing a 5.4% improvement over its training set AUC—indicating robust and stable discriminatory ability in distinguishing between patients with and without AF recurrence. Given the imbalanced outcome distribution, we further generated PR curves to evaluate model performance. In both training and validation sets (Fig. 2A, Fig. 2B), the RF model exhibited the steepest PR curves and the highest area under the PR curve, The PR-AUC of the training set and the validation set were 0.796 and 0.875, respectively, confirming its superior ability to identify patients at risk of AF recurrence compared to the other models.

**Fig. 3:**
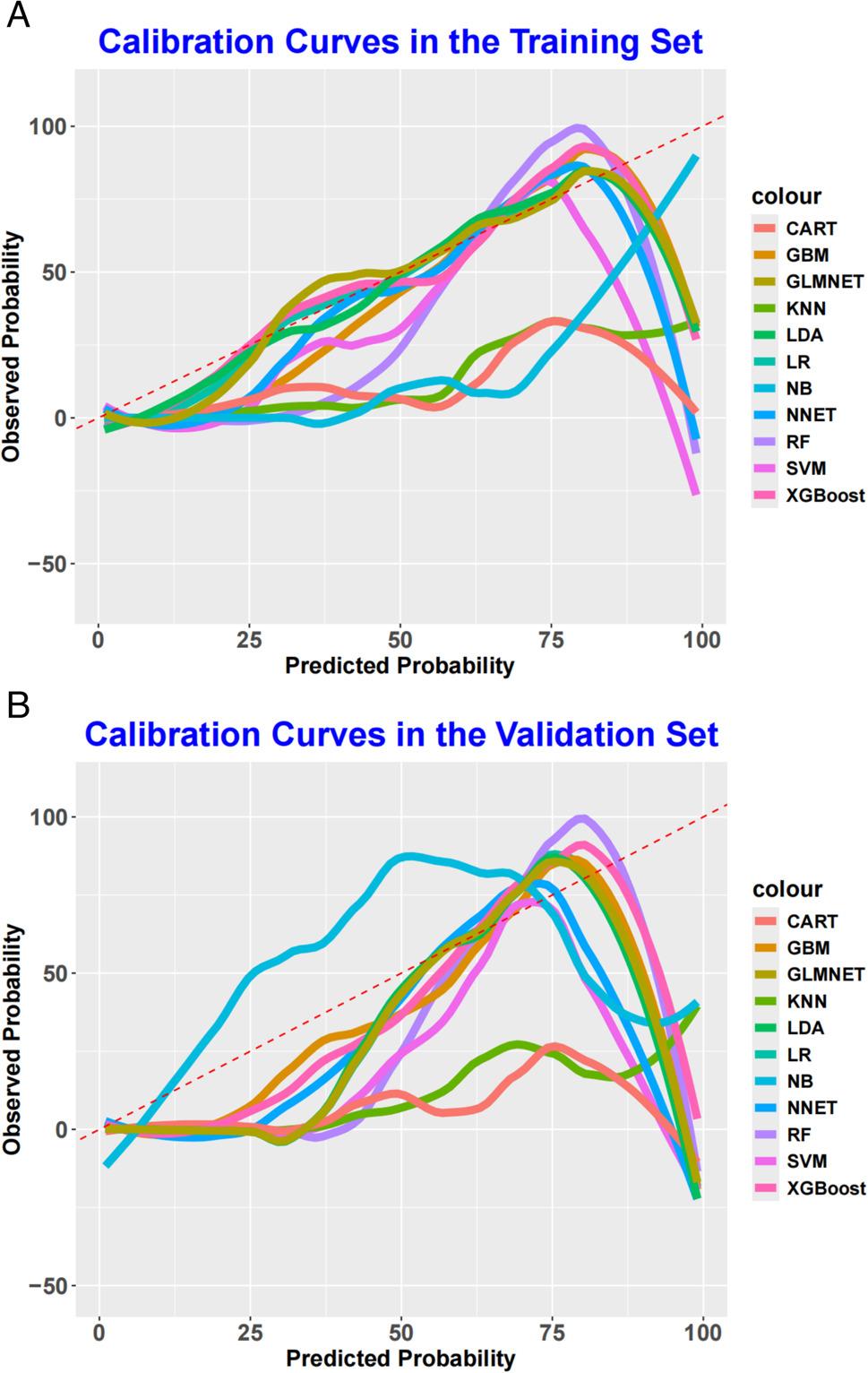
Calibration curve analysis of eleven ML algorithms in the training set (A) and validation set (B)

Calibration curves and Brier scores were used to evaluate the accuracy of predicted probabilities against observed outcomes. The calibration curves (Fig. 3A, Fig. 3B) showed that the RF model’s predicted probabilities aligned closely with the ideal 45° line in both training and validation sets, with no systematic over- or underestimation. In contrast, models such as NNET and KNN showed notable deviations—for instance, NNET overestimated recurrence risk in high-risk patients within the validation set. The RF model also achieved the lowest Brier scores: 0.186 in the training set and 0.192 in the validation set, outperforming traditional models like LR (Brier scores: 0.231 and 0.245, respectively) and other machine learning algorithms, further affirming its excellent calibration. The Brier scoring results of the training set and the validation set are shown in Table 3.

**Table 3:** Brier scores for the training and validation sets of 11 machine learning models

Machine learning model	Training set Briers Score	Validation set Briers Score
LR	0.187	0.201
RF	0.160	0.142
GBM	0.183	0.174
SVM	0.176	0.179
KNN	0.168	0.185
NNET	0.187	0.197
CART	0.175	0.180
XGBoost	0.177	0.186
NB	0.258	0.233
LDA	0.187	0.202
GLMNET	0.187	0.202

**Fig. 4:**
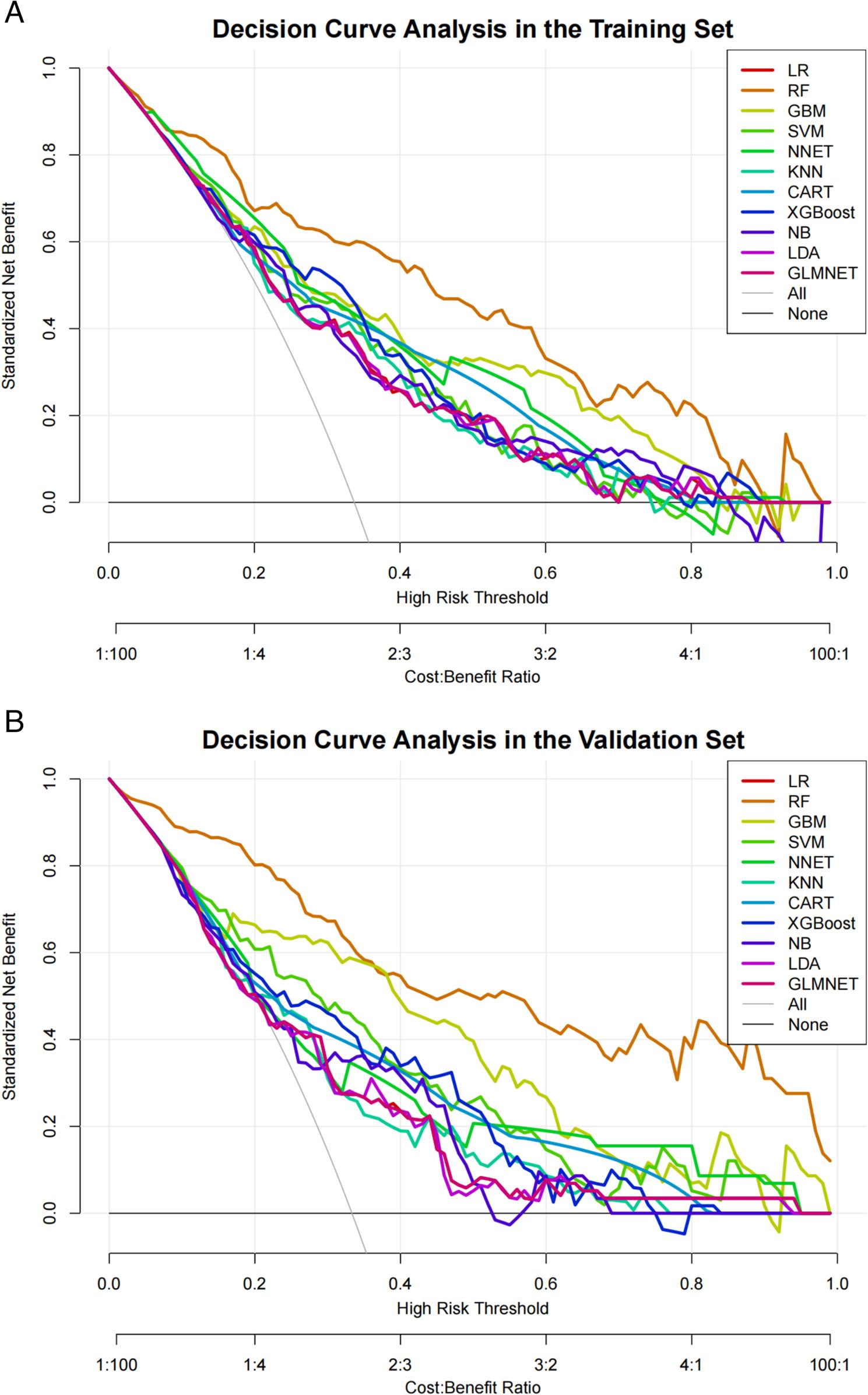
DCA curve analysis of eleven ML algorithms in the training set (A) and validation set (B)

We assessed clinical utility using DCA. In the both training set and validation set (Fig. 4A, Fig. 4B), across a clinically relevant threshold probability range of 0.1–0.7—which guides decisions regarding intensive follow-up—the RF model yielded a higher net benefit than both the “intervene in all patients” and “intervene in no patients” strategies. At the optimal risk threshold of 0.35, the RF model provided a net benefit of 0.42 in the training set and 0.38 in the validation set, the latter being 0.15 higher than that of the LR model. These results suggest that the RF model can effectively identify high-risk patients who may benefit from more intensive post-ablation monitoring, thereby improving clinical decision-making.

**Fig. 5:**
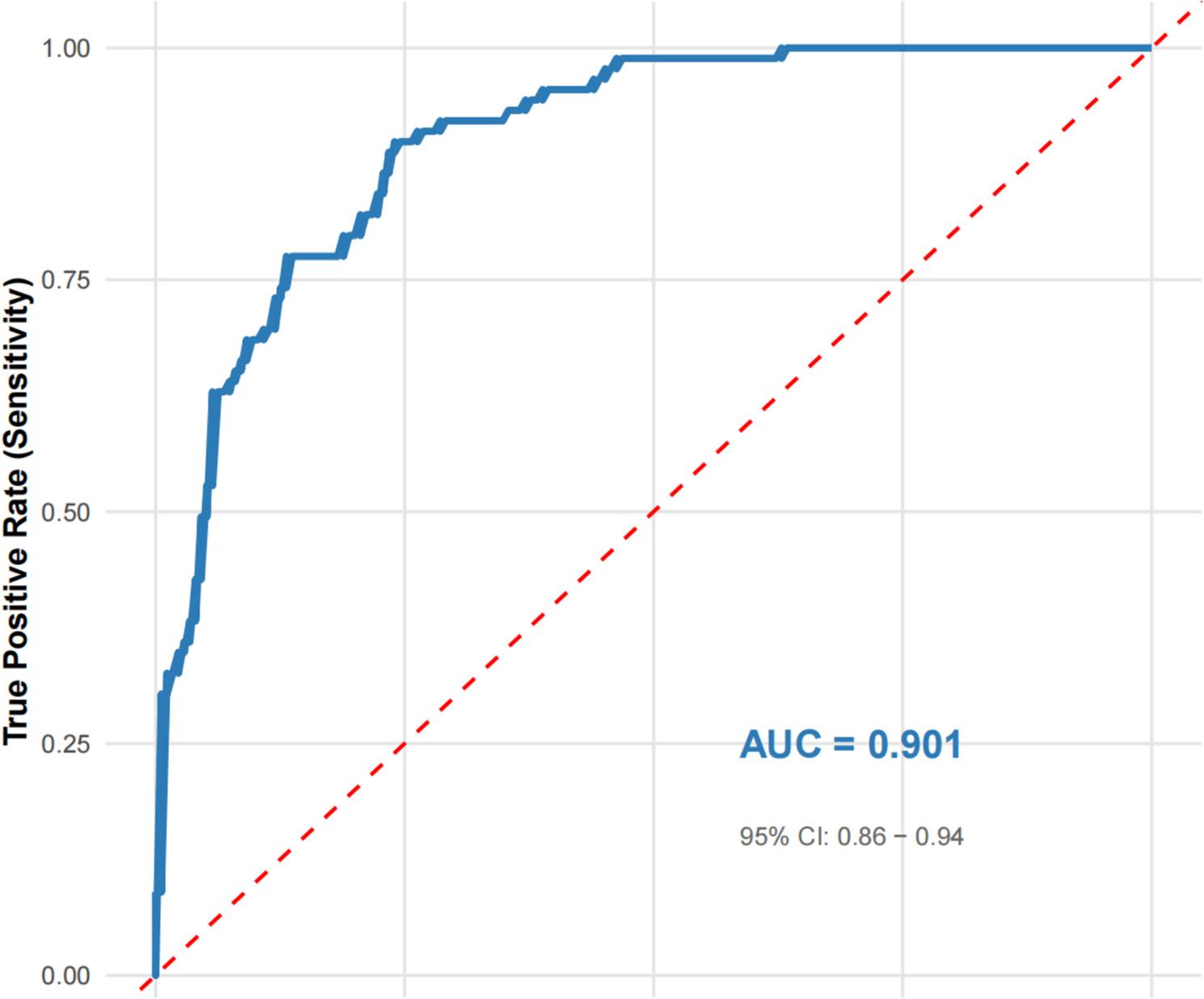
Bootstrap validation of the RF model

**Fig. 6:**
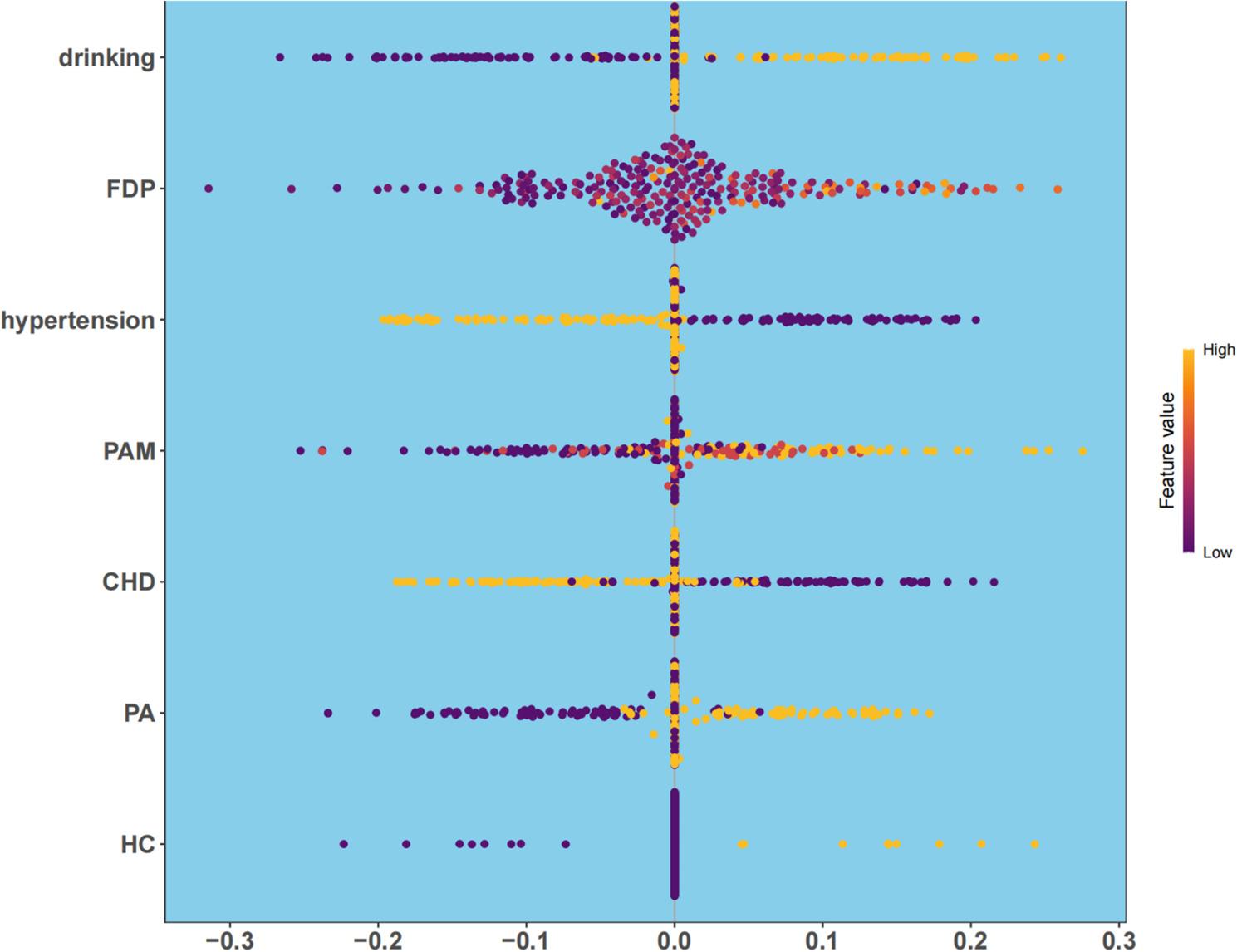
SHAP analysis in RF model

Bootstrap validation with 438 resamples confirmed the robustness of the RF model, yielding a stable AUC around 0.901(Fig. 5), consistent with results from the training and validation sets, indicating no substantial overfitting. To interpret the model, we computed SHAP values (Fig. 6). The top five predictors of AF recurrence, in descending order of importance, were: drinking status (SHAP range: 0.1–0.3), fibrin degradation products (FDP; 0.05–0.25), hypertension (–0.2–0.0), preoperative use of amiodarone (0.0–0.2), and coronary heart disease (CHD; − 0.15–0.05). These findings align with multivariate logistic regression results (such as drinking: OR = 2.12, *P* = 0.017; PA: OR = 2.03, *P* = 0.019), enhancing the clinical interpretability and credibility of the RF model. 

**Fig. 7:**
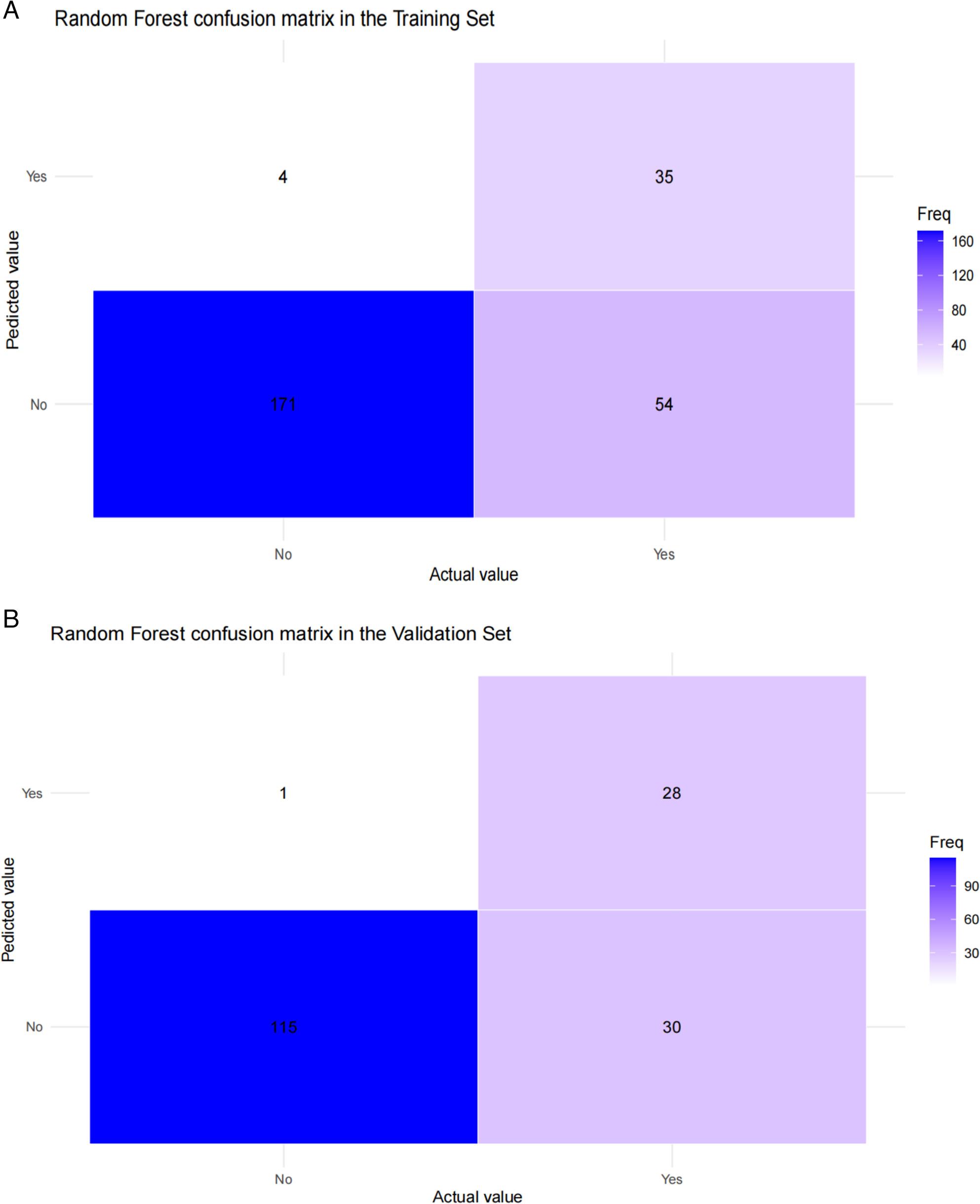
Random Forest confusion matrix in the training set (A) and validation set (B)

Finally, we evaluated classification performance using confusion matrices at a probability threshold of 0.5. In the training set (Fig. 7A), the RF model achieved a sensitivity of 0.816 (71/87), specificity of 0.852 (150/176), an F1 score of 0.783, and a positive predictive value (PPV) of 0.753. In the validation set (Fig. 7B), it maintained robust performance, with a sensitivity of 0.783 (47/60), specificity of 0.831 (98/117), an F1 score of 0.754, and a PPV of 0.723, demonstrating consistent ability to identify true recurrence cases while minimizing false positives across both cohorts.

### Development of nomogram for traditional logistic regression model

**Fig. 8:**
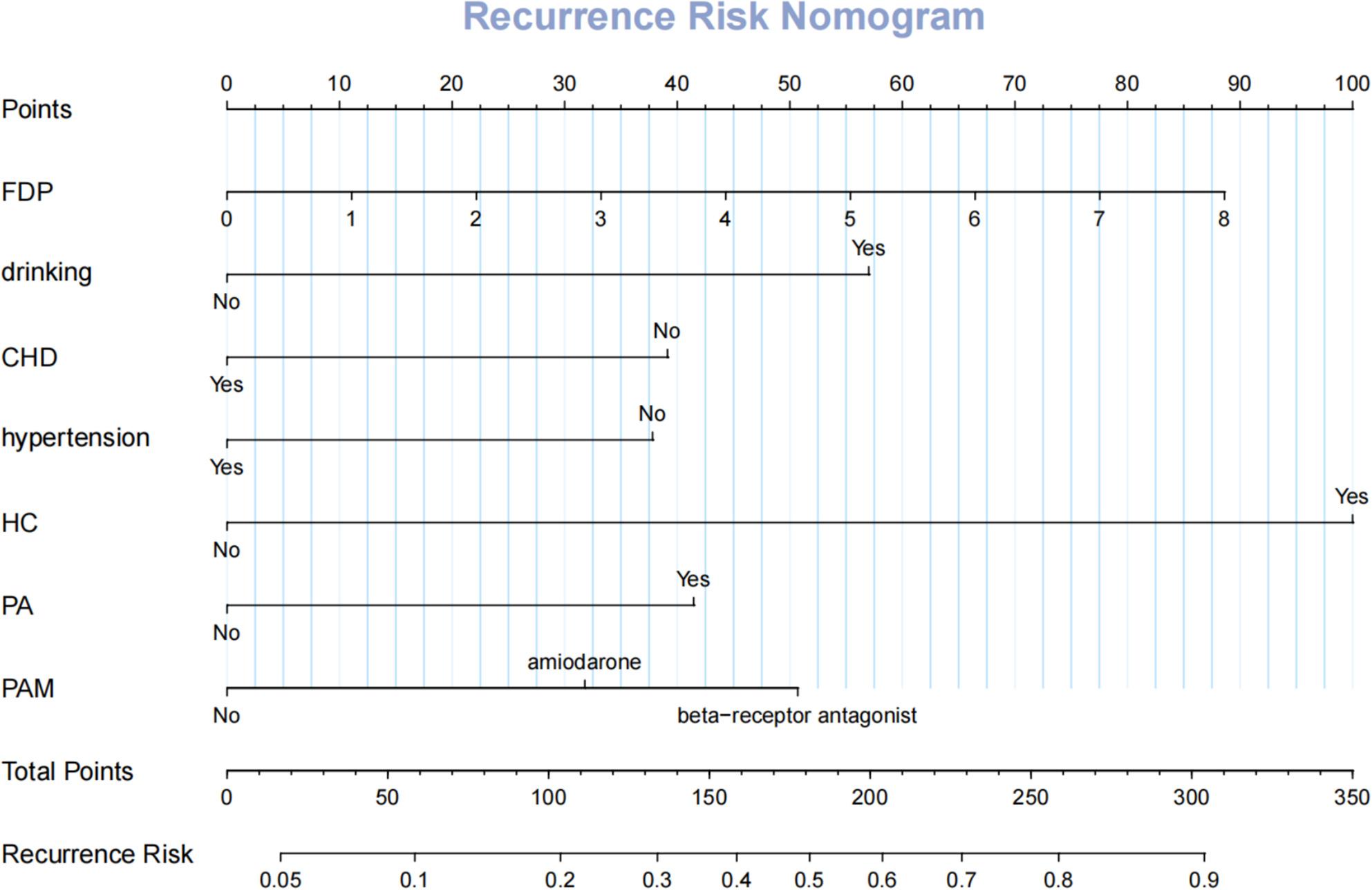
Nomogram for the risk of NAFLD for populations with hypertension

In light of the robust performance exhibited by the traditional logistic regression model in prior analyses, a nomogram was consequently constructed based on five identified risk factors. Through the integration of these risk factors, the nomogram facilitates a more accurate assessment of the probability of specific outcomes (Fig. 8).

## Discussion

In this single-center, retrospective study, we developed and validated a ML-based predictive model for atrial fibrillation recurrence following catheter ablation. By integrating a comprehensive set of clinical, procedural, and laboratory variables, we demonstrated that the RF algorithm outperformed ten other machine learning models and traditional statistical methods. The RF model exhibited exceptional discriminative ability, with an AUC of 0.827 in the training set and 0.925 in the validation set, along with superior calibration and significant net clinical benefit across a wide range of decision thresholds. Beyond outperforming traditional statistical approaches, this model integrated a broad spectrum of demographic, clinical index, laboratory variables, enabling individualized risk prediction that may guide clinical management.

Compared with established risk scores such as CHA₂DS₂-VASc and HATCH, which were designed primarily for thromboembolic and bleeding risk estimation rather than arrhythmic outcomes, the ML-based approach provided substantially greater predictive power for recurrence [17, 28, 29]. This observation is consistent with prior reports indicating the limited utility of these scores in the context of post-ablation AF recurrence [30, 31]. Furthermore, while previous ML studies were often constrained by small cohorts, heterogeneous data quality, and insufficient validation, the current analysis systematically evaluated 11 algorithms in a relatively large real-world cohort and applied bootstrap resampling to confirm model robustness [32]. These methodological strengths add credibility to our findings and provide evidence supporting the integration of artificial intelligence in arrhythmia management.

Beyond predictive performance, this study provides mechanistic insight into recurrence risk by identifying key predictors through SHAP. The SHAP analysis identified the top five predictors of AF recurrence as: drinking status, fibrin degradation products, hypertension, preoperative use of amiodarone, and CHD. These findings were quantitatively supported by the multivariate logistic regression model, which showed that drinking (OR = 2.43, *P* = 0.010) and Preoperative use of Amiodarone (OR = 2.08, *P* = 0.029) were associated with increased recurrence risk, whereas hypertension (OR = 0.48, *P* = 0.028) and CHD (OR = 0.43, *P* = 0.011) were associated with reduced risk. The association of elevated FDP with recurrence may be explained by the interplay between coagulation and inflammation. A pro-thrombotic state can promote a pro-inflammatory milieu, which in turn drives the atrial structural and electrical remodeling that sustains AF [33]. Thus, FDP likely serves as a biomarker of a persistent pro-arrhythmic substrate [34]. The consistency between SHAP importance rankings and regression effect sizes to reinforce the robustness of these predictors and enhances the clinical interpretability of the model [18]. While the predictive utility of the model is clear, the clinical interpretation of some predictors warrants careful consideration. Specifically, the strong association between preoperative amiodarone use and recurrence likely serves as a proxy for underlying disease severity, as this drug is typically reserved for patients with more persistent or refractory AF, a population known to be at higher risk of recurrence post-ablation [35]. Similarly, the seemingly protective associations of hypertension and coronary heart disease may reflect more intensive post-procedural management and risk factor control in these patients, potentially mitigating their baseline risk [36]. These observations highlight that our model captures not only direct risk factors but also complex clinical phenotypes and management patterns. Therefore, while these variables contribute significantly to accurate risk stratification, their associations should be interpreted as reflections of the multifaceted clinical profile of the patient rather than as direct causal mechanisms.

Alcohol consumption emerged as the most influential variable, with an odds ratio of 2.12 (*P* = 0.017), reinforcing prior evidence that alcohol promotes atrial electrical and structural remodeling, autonomic nervous system fluctuations, and direct atrial toxicity [37]. Elevated FDP levels (OR = 1.22, *P* = 0.027) were also significantly associated with recurrence, suggesting that a persistent pro-thrombotic and inflammatory milieu may contribute to atrial remodeling post-ablation [38]. A history of hypertension is associated with a reduced risk of recurrence after atrial fibrillation (OR = 0.47, *P* = 0.012), a paradoxical finding that may reflect more aggressive risk factor control. Likewise, the role of preoperative amiodarone use likely reflects patient selection, as it is often prescribed to those with more advanced arrhythmia burden [39].

By accurately identifying patients at high risk of recurrence, the RF model can inform more individualized decision-making across the care continuum. For instance, patients predicted to have a high recurrence probability could undergo enhanced pre-procedural counseling, consideration of more extensive ablation strategies, or more intensive post-ablation monitoring, while those identified as low-risk could be followed less aggressively, optimizing resource allocation. Decision curve analysis confirmed that the RF model offers superior net clinical benefit across relevant risk thresholds. Furthermore, the development of an interpretable nomogram based on the RF-derived predictors provides a user-friendly bedside tool, enabling clinicians to estimate recurrence risk without immediate reliance on advanced ML software.

The machine learning prediction model developed in this study can be integrated into a holistic management strategy for atrial fibrillation, particularly when aligned with the ABC pathway (A: Anticoagulation/Avoid stroke; B: Better symptom management; C: Cardiovascular and comorbidity management) to optimize patient care throughout the clinical journey [40]. By accurately identifying patients at high risk of recurrence, the model can help tailor anticoagulation decisions in the A component, individualize follow-up and symptom monitoring plans in the B component, and highlight specific modifiable risk factors and comorbidities—such as alcohol consumption, elevated fibrin degradation products, and hypertension—that warrant intensified management in the C component. Thus, the model serves not merely as a standalone predictive tool but can be embedded within a structured AF management framework to enable more precise, comprehensive, and patient‑centered care.

Several strengths of this study should be emphasized. First, the integration of a comprehensive dataset spanning demographic, comorbidity, echocardiographic, and laboratory parameters provided a broad foundation for model development. Second, systematic comparison across 11 ML algorithms ensured unbiased identification of the optimal predictive approach. Third, the incorporation of SHAP values enhanced interpretability, bridging the gap between algorithmic output and clinical reasoning. Finally, internal validation using bootstrap resampling confirmed model stability, mitigating the risk of overfitting. Furthermore, despite starting with a comprehensive set of variables, the application of a stringent variable selection protocol ensured that our final model was based on a parsimonious set of strong predictors, improving its robustness and clinical interpretability.

This study has several limitations. First, the retrospective single-center design may introduce selection bias, and the inability to collect certain important variables—such as detailed procedural parameters and a consistent subclassification of atrial fibrillation—could affect the comprehensiveness and generalizability of the model. Second, although rigorous variable selection was performed to reduce overfitting and a wide range of clinical indicators were included, more refined parameters such as cardiac MRI-based fibrosis and left atrial strain were not incorporated, which may limit the model’s predictive accuracy. Third, recurrence assessment primarily relied on ECG and Holter monitoring; continuous monitoring with an implantable loop recorder might have detected additional asymptomatic recurrences. Despite improved interpretability through SHAP analysis, machine learning models retain a “black-box” nature, which could hinder their clinical adoption. Fourth, the use of a 3-month blanking period to define recurrence differs from the 2-month period recommended in some current guidelines, potentially affecting comparability across studies. Finally, the model is built on baseline data and represents a static risk assessment tool that does not account for dynamic changes in patient risk over time due to treatment adjustments, lifestyle modifications, or new-onset complications. Future studies should validate the model using multicenter, prospective, and long-term follow-up data and explore the development of dynamic prediction models that incorporate longitudinal information. Finally, despite multivariate adjustments, residual confounding from unmeasured or inadequately captured variables—such as detailed lifestyle factors, psychosocial stress, or subclinical inflammation—may influence the observed associations. This underscores that identified predictors should be interpreted primarily from a predictive utility perspective rather than as definitive causal factors.

## Conclusion

In conclusion, this study developed and validated an interpretable RF-based ML model that accurately predicts AF recurrence after catheter ablation. By outperforming traditional scores and other ML algorithms, and by offering both predictive accuracy and mechanistic interpretability, the model holds substantial promise for improving patient selection, guiding tailored follow-up, and advancing precision medicine in the management of atrial fibrillation. 

## Data Availability

Due to privacy and ethical constraints, the data used in the current study are not publicly available, but the data presented in this study are available on request from the corresponding author.
